# Evolution of Codon Usage Bias in Diatoms

**DOI:** 10.3390/genes10110894

**Published:** 2019-11-06

**Authors:** Marc Krasovec, Dmitry A. Filatov

**Affiliations:** Department of Plant Sciences, University of Oxford, Oxford OX1 3RB, UK; dmitry.filatov@plants.ox.ac.uk

**Keywords:** codon usage, phytoplankton evolution, diatoms, effective population size, codon bias

## Abstract

Codon usage bias (CUB)—preferential use of one of the synonymous codons, has been described in a wide range of organisms from bacteria to mammals, but it has not yet been studied in marine phytoplankton. CUB is thought to be caused by weak selection for translational accuracy and efficiency. Weak selection can overpower genetic drift only in species with large effective population sizes, such as *Drosophila* that has relatively strong CUB, while organisms with smaller population sizes (e.g., mammals) have weak CUB. Marine plankton species tend to have extremely large populations, suggesting that CUB should be very strong. Here we test this prediction and describe the patterns of codon usage in a wide range of diatom species belonging to 35 genera from 4 classes. We report that most of the diatom species studied have surprisingly modest CUB (mean Effective Number of Codons, ENC = 56), with some exceptions showing stronger codon bias (ENC = 44). Modest codon bias in most studied diatom species may reflect extreme disparity between astronomically large census and modest effective population size (*N*_e_), with fluctuations in population size and linked selection limiting long-term *N*_e_ and rendering selection for optimal codons less efficient. For example, genetic diversity (pi ~0.02 at silent sites) in *Skeletonema marinoi* corresponds to *N*_e_ of about 10 million individuals, which is likely many orders of magnitude lower than its census size. Still, *N*_e_ ~10^7^ should be large enough to make selection for optimal codons efficient. Thus, we propose that an alternative process—frequent changes of preferred codons, may be a more plausible reason for low CUB despite highly efficient selection for preferred codons in diatom populations. The shifts in the set of optimal codons should result in the changes of the direction of selection for codon usage, so the actual codon usage never catches up with the moving target of the optimal set of codons and the species never develop strong CUB. Indeed, we detected strong shifts in preferential codon usage within some diatom genera, with switches between preferentially GC-rich and AT-rich 3^rd^ codon positions (GC3). For example, GC3 ranges from 0.6 to 1 in most *Chaetoceros* species, while for *Chaetoceros dichaeta* GC3 = 0.1. Both variation in selection intensity and mutation spectrum may drive such shifts in codon usage and limit the observed CUB. Our study represents the first genome-wide analysis of CUB in diatoms and the first such analysis for a major phytoplankton group.

## 1. Introduction

The redundancy of the genetic code is due to multiple codons which encode the same amino acid. The usage of synonymous codons is non-random in most organisms studied so far, with one of the codons being used preferentially—the so-called codon usage bias (CUB) [1,2]. The extent of CUB varies between species and among genes in the same genome. The analyses in the model bacterium *Escherichia coli* in 1980 revealed that more actively expressed genes have stronger CUB [3]—a finding that was later confirmed in *Drosophila* and other species [4]. The debate about the causes of CUB has favored two selective explanations—translational accuracy [5,6,7] and efficiency [8,9], though biases in underlying mutational patterns can also affect or mask CUB [10]. Regardless of the underlying molecular mechanism, the codons that are more frequent in genes with stronger codon bias are typically referred to as ‘preferred’ or ‘optimal’ [11]. In the analyses and discussion presented below, we follow this conventional definition of optimal codons.

The comparisons of CUB between species showed that GC-ending codons are often, but not always preferred to AT-ending codons [12]. For example, a study in 12 species of *Drosophila* showed that the optimal codons are generally GC-rich, except for one species where the optimal codons are AT-rich [13]. The causes for such shifts in optimal codons and CUB are not known, but they may be related to both the shifts in selective pressure and underlying mutational patterns. Three possible factors have been proposed: (i) a small effective population size which changes the relative balance between mutation bias and selection; (ii) a variation in the abundance of tRNAs that changes the pattern of selection mediated by tRNAs; and (iii) a variation of the mutation bias [13]. Mutations may impact the GC content of a genome by decreasing (in many species, e.g., humans, *Drosophila*, yeast or *Arabidopsis* [14,15,16,17,18]) or increasing it (reported in few bacteria, see Table 2 in reference [15]).

Although selection for optimal codons is considered the most plausible explanation for CUB [1], this selection appears quite weak, as hardly any codon bias is detectable in species with a small effective population size, such as mammals [19,20]. The analyses of selection for CUB in bacteria [10] and *Drosophila* [21] indicate that selective coefficients associated with optimal codons are of the order of 10^−6^. This means that selection for optimal codons can overpower drift only in sufficiently large populations with effective population size of the order of >10^6^.

The CUB is relatively well studied in model species, but a large part of the tree of life is not explored. Particularly, phytoplankton, that includes a diverse group of species, including some species with global distribution [22], is an interesting model because population sizes can be astronomically large, which implies very effective selection for weakly selected traits, such as CUB. Given the large population sizes, we expect that CUB in phytoplankton species should be strong, though this prediction has not yet been tested in any phytoplankton species. In this study, we focus on the diatoms, from the Stramenopile kingdom, a major phytoplankton group including 200,000 species [23,24]. We used the transcriptomes of 83 diatom strains representing 36 genera from 4 classes to describe CUB in this major eukaryotic phytoplankton group.

## 2. Materials and Methods

### 2.1. Data Collection

We used the published transcriptomes of the two diatom species *Phaeodactylum tricornutum* RCC2967 [25] and *Thalassiosira pseudonana* CCMP1335 [26], and the database of the Marine Microbial Eukaryote Transcriptome Sequencing Project (MMETSP) [27] that includes 81 diatoms transcriptomes available at https://www.imicrobe.us/#/projects/104. Two of the studied species—*Aulacoseira subarctica* (MMETSP1064) and *Thalassiosira* sp. (MMETSP1059)–come from freshwater, while the remaining 81 species and strains analysed come from marine environments all over the world, including samples from open ocean and coastal ecosystems, and ranging in optimal growth temperature from 2 to 29 °C. In total, the dataset includes 67 species from 35 genera belonging to 4 diatom classes. The details of the species and strains used in the analyses are listed in Table S1.

### 2.2. Codon Usage Analysis

Codon usage analysis was done with CodonW (http://codonw.sourceforge.net), with each species analysed separately. Before analysis, we removed genes with internal stop codon(s) in the reading frame and genes shorter than 200 codons to reduce variance due to small samples. The number of genes analysed varied substantially between species, ranging from 754 to 25,018 with an average of 11,185 genes per species. First, we ran a correspondence analysis to generate the files containing the preferred and unpreferred codons (based on a 2-way Chi squared contingency test). Then, we calculated the frequency of optimal codons (FOP) and the effective number of codons (ENC) gene by gene and with the total transcriptome. Additional analysis allowed us to extract the total GC content and the GC content at GC3s sites of the whole transcriptome. As selection toward optimal codons is thought to be stronger in highly expressed genes, we also calculated the ENC, FOP and GC3s of the genes with higher and lower expression (defined as 500 genes with the highest and 500 genes with the lowest expression in each species). For strains from MMETSP project we calculated expression values (FPKM) from the counts of reads mapped to each transcript that are available from https://www.imicrobe.us/#/projects/104. To obtain expression values for *P. tricornutum*, the previously published RNAseq data [28] (SRA numbers: SRS3629289, SRS3629290 and SRS3629291) were aligned against the reference transcriptome of this species using RSEM v.1.2.31 [29] (standard parameters). Correlation analysis and statistical tests between the different indexes were done with R v3.5.1 [30].

### 2.3. Analysis of Divergence within Genera

To estimate divergence between species and strains of the same genus, we first identified orthologous genes using orthofinder v2.2.6 [31] with standard parameters for each genus with multiple species (>5) or two strains of a same species: *Chaetoceros* (9 species or strains), *Pseudo-nitzschia* (8 species or strains), *Skeletonema* (11 species or strains) and *Thalassiosira* (12 species or strains), *Leptocylindrus danicus* (3 strains), *Minutocellus polymorphus* (3 strains), *Asterionellopsis glacialis* (2 strains) and *Fragilariopsis kerguelensis* (2 strains). Single-copy orthologs in the same genus were aligned using PRANK v140110 (options: -showevents -showanc -codon -DNA) [32] and nucleotide variation at silent sites was calculated with SNAP v2.1.1 [33]. The output file from PRANK gave the substitution events on each branch of the genus phylogeny. These substitutions were used to analyse synonymous codon changes from the ancestral sequence. The ancestral sequence was inferred by maximum likelihood using BPPAncestor from the BPPSuite package [34]. The orthologous genes were then used for phylogenetic tree reconstruction in each genus using RAxML v7.2.8 [35] with GTR Gamma and 1000 bootstraps. To increase the number of analysed substitutions per branch, we only looked at the substitution pattern at the species level. In the case of several strains for one species, we randomly kept only one as follows: in *Skeletonema*, we kept *Skeletonema marinoi* FE60 (MMETSP1040) to represent all *S. marinoi* strains, except *S. marinoi*_UNC1201 (MMETSP1428) that appears more distant from the group (see below). In *Thalassiosira*, we kept just one *Thalassiosira rotula* (MMETSP0404) and one *Thalassiosira weissflogii* (MMETSP0881). Due to the very low divergence between *Thalassiosira* sp FW (MMETSP1059) and *T. pseudonana* CCMP1335 (see below), we only kept MMETSP1059.

We ran a full orthofinder analysis with all (83) strains included. This generated a STAG (Species Tree inference from All Genes) phylogeny inferred from all orthogroups and rooted using STRIDE (Species Tree Root Inference from gene Duplication Events) [36,37]. Briefly, orthofinder builds a multiple sequence alignment for each orthogroup with MAFFT [38] and creates gene trees with IQTree [39] using Maximum-Likelihood. Then, STAG infers a tree from each set of orthologs from orthofinder run with shortest distance pairs genes by analysing each pair of sequences. That permits to increase the data availability by using all orthogroups and not only the one-to-one orthologs. Then, the STAG tree is rooted with STRIDE through the identification of gene duplication events. STRIDE avoids the need for an external outgroup and eliminates the problem of long-branch attraction effect [37].

### 2.4. Species Delimitation

For genera with multiple (>5) strains sequenced (*Chaetoceros*, *Pseudo-nitzschia*, *Skeletonema* and *Thalassiosira*), we used a statistical approach to establish and clarify the species/strains assignments. We used BPP, a Bayesian Markov chain Monte Carlo software, for analysing genomic data under the multispecies coalescent model [40,41]. We ran the A11 analysis (Species-delimitation: 1 and Species-tree: 1) to explore different species assignations and phylogenetic trees and identify the best species/strains grouping. We used the previously reconstructed RAxML trees as the required input (*species&tree* option in the *bpp.control.ctl*) file.

### 2.5. Estimation of the Strength of Selection for Optimal Codons

Following Bulmer [2] and Sharp and colleagues [10], we estimated the strength of selection for CUB from the frequency of optimal codons, assuming the observed frequency is at mutation-selection-drift equilibrium. Let us consider the preferred codon C1 and unpreferred codon C2 with mutation rate from C1 to C2 equal *u* and C2 to C1 equal *v*. Assuming the fitness of the optimal codon C1 is 1, the fitness of C2 is 1−*s* with *s* denoting the fitness difference between C1 and C2. Then, the frequency of optimal codons (*P*) under selection-mutation-drift equilibrium is:

*P* = *e^s^V*/(*e^s^V* + *U*) (1)

where *S* = 2*N*_e_*s*, *U* = 2*N*_e_*u* and *V* = 2*N*_e_*v*, with *N*_e_ standing for effective population size. From this, the strength of selection can be estimated as (with *k* = U/V):

*S* = ln[(*P* × *k*)/(1 − *P*)] (2)

In *P. tricornutum*, the mutation spectrum is known [42], so *k* is readily available. In the case of GC-rich preferred codon, *u* = 2.21 and *v* = 1.0, so *k* = 2.21. In the case of AT-rich preferred codon (*u* = 1.0, *v* = 2.21 and *k* = 0.45), which allows us to calculate *S* for this species from the observed *P*. For other diatom species, where mutation spectrum has not been studied experimentally, we followed the approach of Sharp and colleagues [10] to assume that for weakly expressed genes selection on codon usage is negligible, so Equation (1) reduces to *P = V/(V + U)*, which allows us to estimate *k* for each species:

*k* = (1 − *P*)/*P* (3)

Assuming the same *k* is shared by all genes in a species, we calculated *S* for strongly expressed genes from Equation (2).

To test whether there is significant evidence for positive selection on CUB, we followed the method of Sharp and colleagues [10] that calculated *S* in random sets of genes (here 1000 sets of 500 genes) and compared the resulting distribution to the *S* calculated for the 500 highly expressed genes. *S* for the randomly selected gene sets were calculated with *k* from the 500 lower expressed genes. If *S* of the highly expressed genes was in the 95% or 99% upper tail of the distribution of S values from random genes, we concluded that *S* in top-500 highly expressed genes is significantly higher than for the rest of the genome.

## 3. Results

### 3.1. Codon Bias in Diatoms

In the absence of codon bias, all synonymous codons are expected to be used at random, while with an extreme codon bias, only 20 codons would be used, one per each amino acid. Thus, the extent of codon bias is often measured with the Effective Number of Codons (ENC [43]) which ranges from 61 (no bias) to 20 (maximal codon bias). Other measures of codon bias, such as Frequency of Optimal Codons (FOP [11]) and proportion of G or C nucleotides at the 3^rd^ codon position (GC3) are also commonly used. The genome-wide average values of ENC, FOP and GC3s indexes for all 83 diatom species and strains analysed are listed in Table S2 and the distributions of indexes per gene are shown in Figure S1. The strongest codon usage bias (CUB) was observed in *Chaetoceros curvisetus* (ENC = 44.7; FOP = 69.1%), while most other species had considerably weaker CUB, with mean ENC = 56.02. Consistent with analyses in other organisms [3,4], stronger CUB in diatoms was observed for the more actively expressed genes: on average, ENC showed 4.55 points difference between 500 most- and 500 least-actively expressed genes (Table S2).

**Figure 1:**
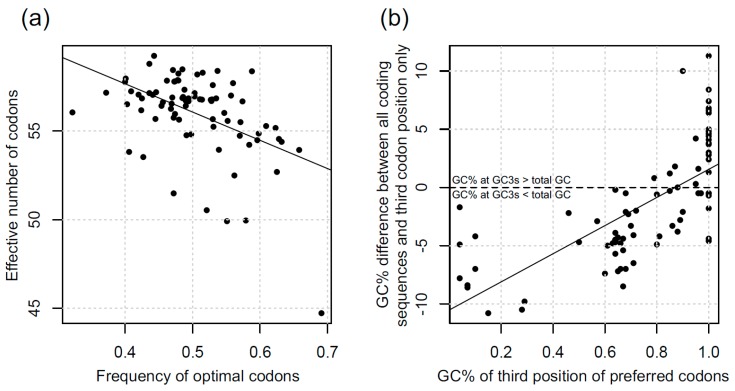
(**a**) Correlation between effective number of codons and frequency of optimal codons considering all genes in each species. Pearson’s correlation: rho = −0.475, *P* value = 6^E-6^; and (**b**) Correlation between the GC% of the optimal codons’ third position and the difference of GC% between all the coding genome and GC3s only. Pearson’s correlation: rho = 0.695, *P* value = 3^E-13^.

The indexes show strong variation in CUB within some genera (e.g., *Chaetoceros* contains strains with the strongest and weakest CUB, Table S2), while other genera show very similar CUB across the strains (e.g., in *Skeletonema* ENC = 56.77 ± 0.13). To some extent, this reflects the divergence between the species in the genus, as *Skeletonema* strains show the lowest pairwise synonymous divergence compared to other genera (see below). The strains with strongest CUB (lower ENC at actively expressed genes) are spread across the genera and do not show any clustering in a particular taxonomic group of diatoms. ENC and FOP are highly negatively correlated (Pearson’s correlation: rho = −0.475, *P* value = 6^E-6^, Figure 1a), which is hardly surprising given both indexes reflect the extent of CUB, with strong CUB corresponding to higher FOP and lower ENC.

The optimal codons (Tables S3 and S4) show a strong variation of the GC content at the third position across the strains. GC content at the 3rd codon position of optimal codons ranges from 4% to 100% GC. Three subsets of strains are apparent in the data: the strains with AT-rich optimal codons, where the GC3s of optimal codon only is between 4% and 29% (10 strains); the species with moderately GC-rich optimal codons with GC3s between 46% and 72% (32 strains); and the strains with highly GC-rich optimal codons, where GC3s is between 79% and 100% (41 strains). In this last group, 23 strains have a GC content of 100% at the GC3s position of optimal codons. These results show that the optimal codons are generally GC-rich in diatoms, but 12% of strains (10 out of 83 including 2 of the 9 *Chaetoceros*) have highly AT-rich optimal codons. Three orders are represented among the 10 AT-rich strains (Fragilariophyceae, Coscinodiscophyceae and Bacillariophyceae), and among the 23 100% GC-rich strains (Bacillariophyceae, Coscinodiscophyceae and Mediophyceae).

We tested the GC content difference between all coding sequences and GC3s sites only (Figure 1b). It appeared that the GC content at GC3s sites, even in several GC-rich optimal codons species, is not necessarily higher than the average GC of coding sequences, suggesting that CUB is, at least partly driven by mutational bias(es). Only a part of the highly GC-rich optimal codon species have a higher GC content at GC3s sites. Last, the GC content at GC3s sites is always lower than the total GC content in the AT-rich species.

Then, we compared the genomic GC content in the two species for which a whole genome is available, *T. pseudonana* CCMP1335 and *P. tricornutum* RCC2967. Both have lower GC content in non-coding sequences than in coding sequences: 46% versus 48% in *T. pseudonana* CCMP1335 and 46% versus 51% in *P. tricornutum* RCC2967. By comparison, the GC3s are 46% and 51% in *T. pseudonana* CCMP1335 and *P. tricornutum* RCC2967, respectively.

### 3.2. The Phylogeny of the Diatom Species Analysed

**Figure 2:**
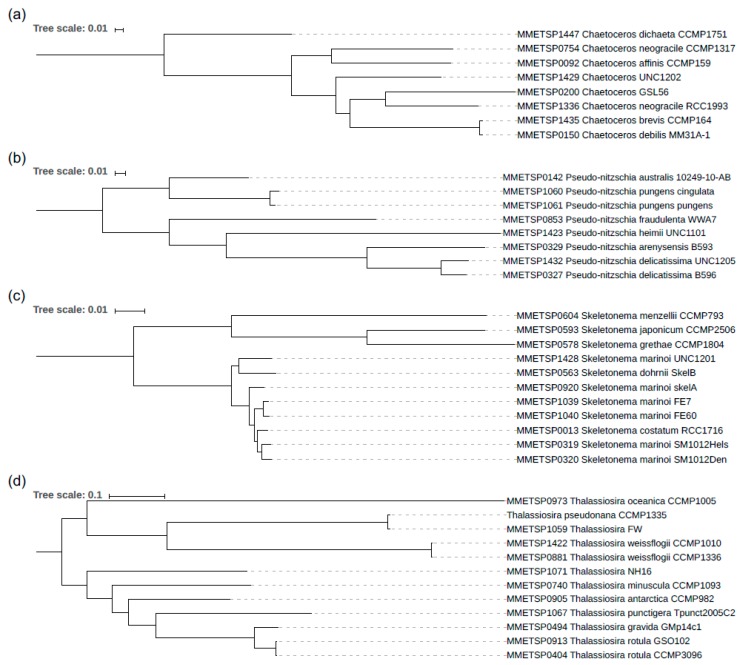
Phylogenies of *Chaetoceros* (**a**); *Pseudo-nitzschia* (**b**); *Skeletonema* (**c**); and *Thalassiosira* (**d**) obtained by RaxML from the single orthologs identified by Orthofinder. DensiTree plots for these genera are shown in Figure S3.

To study whether phylogenetic relationships can account for the patterns of codon bias evolution, we used Orthofinder to identify the 1-to-1 orthologous genes between the transcriptomes analysed. This analysis was conducted for the total dataset as well as separately for *Chaetoceros*, *Pseudo-nitzschia*, *Skeletonema* and *Thalassiosira* genera for which at least 8 strains per genus were available. Orthofinder identified 53 1-to-1 orthologs in *Chaetoceros*, 1090 in *Pseudo-nitzschia*, 125 in *Skeletonema* and 204 in *Thalassiosira*. These orthologs were used to reconstruct phylogenies for each of these genera using RAxML, as described in the methods. The resulting phylogenies of *Chaetoceros*, *Pseudo-nitzschia*, *Skeletonema* and *Thalassiosira* are shown in Figure 2a–d, respectively. Orthofinder did not identify any single orthologs shared by all 83 strains from our dataset, but it did reconstruct a phylogeny with all strains and all orthogroups using STAG approach [36] (Figure S2). Given high divergence between species and strains, the phylogenetic relationships in this overall diatom phylogeny have to be taken with caution.

### 3.3. Species Assignments and Delimitations for the Diatom Strains Analysed

Sequence divergence between the strains of the same genus allowed us to identify likely species- strains mis-assignments in the set of diatom strains analysed (*dS* values provided in Table S5). In particular, while synonymous pairwise divergence (*dS*) between most *Chaetoceros* strains (Figure 2a) vary from 0.59 to 0.77, this value is only *dS* = 0.03 for *Chaetoceros debilis* MM31A-1 and *Chaetoceros brevis* CCMP164, suggesting that MM31A-1 and CCMP164 may belong to the same species. On the other hand, *dS* between the two strains of *Chaetoceros neogracile* (MMETSP1336 and MMETSP0754) is 0.71, indicating that these two isolates actually belong to fairly divergent species. While the two *C. neogracile* strains cannot possibly belong to the same species given 71% divergence, the 3% divergence between MM31A-1 and CCMP164 may correspond to divergence between closely related species or intra-specific polymorphism within the same species. To clarify species delimitations within the genus, we used Bayesian multi-coalescent-based approach implemented in BPP [40,41]. This approach confirms that all 8 *Chaetoceros* strains belong to different species (posterior probability = 0.9998 for 8 species). This species assignment is also supported by multi-genic phylogenies as shown on the densiTree plot on Supplementary Figure S3. 

In genus *Pseudo-nitzschia*, *dS* between the species ranges from 0.42 to 0.71, while *dS* = 0.12 for the two *Pseudo-nitzschia delicatissima* strains and *dS* = 0.03 for the two *Pseudo-nitzschia pungens* strains (Figure 2b). We reason that *P. delicatissima* strains likely belong to different closely related species, while the two *P. pungens* strains might be correctly assigned to the same species. However, the Bayesian species delimitation [40,41] indicates that each of the 8 *Pseudo-nitzschia* strains is a different species (Figure S3), including the two *P. pungens* strains (posterior probability = 0.881730).

**Figure 3:**
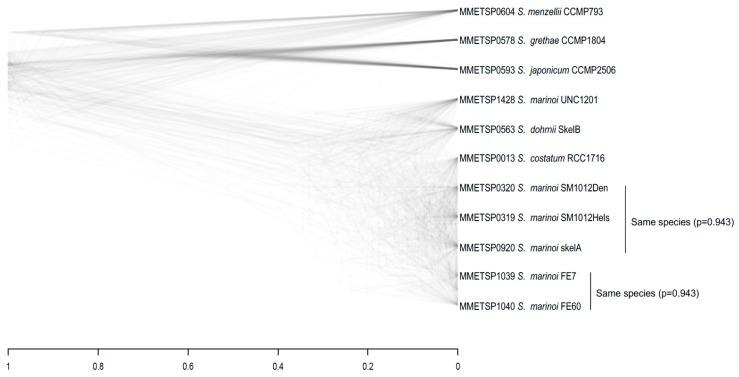
DensiTree of the 125 orthologs in *Skeletonema* genus. The two species assignments come from the A11 analysis with posterior probabilities [40,41].

In *Skeletonema* (Figure 2c), *dS* clearly splits the strains into two categories, one with high divergence (0.20 < *dS* < 0.34), corresponding to interspecific divergence and the second group with 0.01 < *dS* < 0.07 including five *S. marinoi* strains, two *S. costatum* (RCC1716; MMETSP0013) and *S. dohrnii*. In the low-divergence subset, *S. dohrnii* is 7% divergent from other strains, indicating that this strain is correctly assigned to a separate species. However, in two cases, species names appear to be incorrectly assigned to *Skeletonema* strains. First, *S. costatum* (RCC1716) is very close to other *S. marinoi* species (*dS* = 0.02) suggesting they are likely conspecific. Second, *S. marinoi* UNC1201 (MMETSP1428) is divergent from all other *S. marinoi* species with (*dS* = 0.06). The Bayesian species delimitation strongly supports (posterior probability = 0.94329) the assignment of 11 analysed *Skeletonema* strains to 8 species, merging the two strains MMETSP1039 and MMETSP1040 (*dS* = 0.01) and the three strains (MMETSP0013, MMETSP0319 and MMETSP0320 (that have 0.01 < *dS* < 0.02). Multigenic phylogenies shown on *Skeletonema* densiTree plot (Figure 3) support this species assignment. As expected, within the species different genes show different phylogenies, while for separate species gene-specific trees agree with each other.

While most *Thalassiosira* strains (Figure 2d) are highly divergent (0.16 < *dS* < 0.81) and clearly correspond to different species, three pairs of strains show low divergence (*dS =* ~0.01 for pairs CCMP1335 and MMETSP1059; two *T. weissflogii* strains and two *T. rotula* strains) that likely reflects that they are conspecific. The low divergence (*dS* = 0.01) between the model diatom strain *T. pseudonana* CCMP1335 (isolated in the sea) and the fresh water isolate MMETSP1059 is consistent with previous reports that *T. pseudonana* is likely to be a freshwater species with high salt tolerance [44,45]. The BPP A11 analysis of 12 *Thalassiosira* strains gave two high probability species assignments, one with 11 species (merging the two *T. weissflogii* strains; posterior probability = 0.5829), and the other with 10 species (same as with 11 species but also merging the two *T. rotula* strains; posterior probability = 0.4149).

Regarding the strains in the less represented genera in our dataset, the two *A. glacialis* strains show very high divergence (*dS* = 0.52; 4407 single orthologs analysed), indicating they belong to different species. The two *F. kerguelensis* (6129 single orthologs) strains are relatively closely related (*dS* = 0.04), but 4% is likely still too divergent to belong to the same species. The three *L. danicus* strains (2235 single orthologs) show high divergence (*dS* > 0.15), indicating that all three strains belong to different species. On the other hand, two of the *M. polymorphus* strains are quite closely related (*dS* = 0.034 for strains NH13 and CCMP3303), indicating that they might be conspecific, while the third *M. polymorphus* strain (RCC2270) is highly divergent from the other two (*dS* = 0.31), corresponding to interspecific divergence. Too few strains are available for these genera to analyse species delimitation with Bayesian multi-coalescent approach [40,41].

### 3.4. Effective Population Size in Marine Plankton Species

Our analysis revealed that most diatom species have modest CUB, which is surprising given large population sizes of marine plankton species. Why is CUB so modest in most diatom species? A possible reason for this may be that selection for preferred codons may not be strong enough to overpower drift. Despite (presumably) very large census sizes, the effective population sizes for these species may be too small to make selection for preferred codons sufficiently effective. The extreme disparity between very large census population size and relatively small effective population size reported for the non-diatom phytoplankton *Emiliania huxleyi* [46] and *Ostreococcus tauri* [47] may potentially explain why selection in most diatom species is not effective enough to drive CUB to high values. To test this hypothesis, we estimated genetic diversity in two *Skeletonema* species where BPP analysis (in the previous section) assigned more than 1 strain per species. One of these BPP-defined species includes MMETSP1039 and MMETSP1040, while the other includes MMETSP0920, MMETSP0320 and MMETSP0319. Average pairwise per nucleotide diversity at silent sites (*π*) for these species are 0.01 and 0.02, respectively. Assuming that mutation rate in these species is similar to that in *P. tricornum*, i.e., *μ* ~4.77 × 10^−10^, we can estimate effective population size (*N*_e_) from *π* = 4*N*_e_*μ* to be *N*_e_ ~10 million individuals. Similarly, the two *T. rotula*, two *T. weissflogii* and two *S. marinoi* (MMETSP1039 and MMETSP1040) have *π* ~0.01, giving *N*_e_ ~5 million individuals. Furthermore, it was previously reported that several other marine plankton species have *N*_e_ ~10 million: diatom *P. tricornum* [42], coccolithophore *E. huxleyi* [46] and coastal green algae *O. tauri* [47]. Thus, effective population sizes of plankton species analysed so far are of the order of a few million individuals, which indicates the generality of the finding that marine plankton typically have relatively modest *N*_e_, despite astronomically large census population sizes [46]. However, terrestrial organisms with *N*_e_ of a few million individuals, such as *Drosophila*, do have significant codon bias [4,13,21]. Thus, if selective advantage of preferred codons compared to unpreferred ones(*s*) in plankton is similar to that in *Drosophila* (*s* ~10^−7^ to 10^−6^ [48]), *N*_e_ >1 to 10 million in plankton populations should be large enough to render selection on codon usage sufficiently effective to overpower drift. Another way to test whether selection for codon usage is effective in diatoms is to estimate the strength of selected CUB.

### 3.5. Estimating the Strength of Selection for Optimal Codons in Diatoms

Assuming that codon bias is at mutation-drift-selection equilibrium, we can use the approach previously employed for bacteria [10] to estimate the strength of selected CUB from the frequency of optimal codons in diatom species. Using equation 2 (see methods) we estimated the population-scaled selective coefficient, *S* (= 2*N*_e_*s*), for strongly expressed genes (500 most actively expressed genes) in all the diatom strains analysed. The resulting estimates of *S* ranged from −0.88 to 0.91 (Table S4). The estimates for *S* were negative in 22 strains, including all AT-rich preferred codon strains (the 10 species with high AT bias and the moderate AT bias strains MMETSP1065). Furthermore, 3 of the 5 strains with *S* > 0.65 belong to the *Chaetoceros* genus (MMETSP0092, MMETSP1336, MMETSP0150). This analysis included *P. tricornutum* RCC2967, where the mutation rate and spectrum were measured directly in a mutation accumulation experiment [42], which allowed us to compare the estimates of *S* based on mutational pattern observed directly and inferred from equation 3 as was done for all other strains analysed. This comparison revealed that *S*, based on equation 3 (*S* = 0.463), is twofold lower compared to the one based on actual mutational pattern (*S* = 1.1).

Importantly, this approach for estimation of the strength of selected CUB assumes mutation-drift-selection equilibrium [10], which is likely violated in strains where the preferred set of codons has changed relatively recently. This may be particularly problematic for species with AT-rich preferred codons that belong to clades with GC-rich preferred codons as such species must be out of equilibrium, which probably explains the negative estimates of *S*. To minimise this ‘out of equilibrium’ problem we excluded all AT-rich strains and limited the analysis to only codons that did not show any change in preferred codon usage within a clade. Even taking only CG rich codon strains, no codons are preferred in all strains, so we restricted the analysis to a smaller group of strains (Table 1) where universally preferred codons can be found for some of the amino acids with two codons (Asn, Cys, Gln, Glu, His, Lys, and Tyr in all groups; Asp in *Pseudo-nitschia*, *Minutocellus* and *Skeletonema* only; Phe in *Leptocylindrus*, *Thalassiosira* and *Skeletonema* only). With this smaller set of species and codons, the three *Pseudo-nitzschia* species with previously negative *S*, now have *S* > 0, but one strain of *M. polymorphus* still has *S* < 0 (Table 1). The resulting estimates of *S* are relatively modest (all < 1), indicating that selected codon usage bias in diatoms is quite weak. To test whether the calculated *S* are significantly high (i.e., whether there is evidence for selected CUB), we follow the method of Sharp and colleagues [10] that calculated *S* in random sets of genes. We found that our *S* values are significantly higher than that of the randomly selected genes in all cases, excepted for *M. polymorphus* CCMP3303 that has *S* < 0 (Table 1).

### 3.6. Evolution of Codon Bias within Diatom Genera

Modest CUB in most diatom strains analysed may be explained by occasional changes in the set of preferred codons. Evolution of codon bias is a slow process, with actual codon usage slowly approaching the optimal codon usage if selection is sufficiently effective to overpower drift. If the changes in the set of optimal codons occur frequently enough, the actual codon usage never catches up with the moving target of the optimal set of codons and never develops strong codon bias. This idea is supported by the fact that the optimal set of codons differs between the species, even within some of the diatom genera (Table S2). Indeed, the genus *Chaetoceros* includes species with GC-rich and AT-rich optimal codons (*C. dichaeta* CCMP1751 and *C. neogracile* CCMP1317), which likely occurred independently and thus represent at least two major shifts in the set of preferred codons within the same genus. 

More subtle changes in preferred codon usage are also evident from comparisons of species within each of the diatom genera. In *Chaetoceros*, GC-rich species (particularly MMETSP0150, MMETSP0200, MMETSP1336 and MMETSP1429) share some AT-ending preferred codons with the AT-rich species MMETSP1447, but only for amino-acids with 4 or more codons (Leu, Val, Ser, Pro, Thr, Arg and Gly). The GC-rich species in that case have one GC- and one AT-ending preferred codon for 7 amino acids with 4 possible codons. In *Pseudo-nitzschia*, the preferred codon usage also shows some variation. First, the GC-ending preferred codon for the same amino acid shows changes between species (e.g., CUC or CUG for the leucine). Second, three GC-rich species (MMETSP0327, MMETSP0329, MMETSP143) have one AT-ending preferred codon in addition to the GC-ending one for 4 amino-acids (Ile, Pro, Arg and Gly). The same is observed in two *Thalassiosira* species (CCMP1335 and MMETSP1059) for 7 amino acids (Ile, Ser, Pro, Thr, Ala, Arg and Gly) and in MMETSP0905 with Arginine. The *Skeletonema* genus is the most stable in terms of the set of preferred codons, possibly due to more recent species divergence in this genus (Figure 3). The only variation observed between *Skeletonema* species is for the codons encoding serine, where most of the species do not have a preferred codon, while AGU is the preferred codon in MMETSP0013, MMETSP0319 and MMETSP0320. Apart of this, the GC-ending codon is preferred for all amino-acids encoded with two codons. However, for the amino-acids encoded by 3 or more codons, two preferred codons are identified: one GC- and one AT-ending. Such patterns with one AT- and one GC-ending preferred codon in some species may explain the low GC at GC3s sites compared to total the GC in some GC-rich species (Figure 2b), because selection could act both toward GC or AT codons depending on the considered amino acid.

These shifts in the set of optimal codons have to result in the change of the direction of selection for codon usage, preventing the species from reaching mutation-selection-drift equilibrium. The change in underlying mutational patterns may also prevent the species from reaching equilibrium. Indeed, the analysis of substitution patterns in the species branches (the external branches) of the trees (Figure 3) revealed that all the diatom species analysed are out of the equilibrium GC content at the 3rd codon positions (Table 2). Note that this analysis is focusing on species rather than strains; that is, only one strain per species is included (Table 2). The *Chaetoceros* species with GC-rich preferred codons shows a highly significant increase of the GC content (at least 15%) at third codon position compared to the ancestral sequence reconstructed with maximum likelihood (as detailed in the methods). On the other hand, one of the two *Chaetoceros* species with AT-rich preferred codons (MMETSP0754) increased AT-content by ~18%, while the other AT-rich species (MMETSP1447) decreased AT-content by ~2%. Interestingly, the two strains of *C. neogracile* show strong difference in CUB: one of these strains has AT-rich preferred codons (*C. neogracile* CCMP1317 MMETSP0754 with GC content of 15% at third position of preferred codons), while the other one has GC-rich preferred codons (C. *neogracile* RCC1993 MMETSP1336 with GC content of 61% at third position of preferred codons). However, as explained above, these two C. *neogracile* strains are quite divergent (*dS* = 0.71) and must belong to different species. In *Skeletonema*, where all species have mostly GC-rich preferred codons (GC3 of preferred codons from 63% to 67%), the GC content increases in all species. Within *Pseudo-nitzschia* and *Thalassiosira* genera, the analysis of substitutions showed mixed patterns (Table 2), with some species showing an enrichment in GC at silent sites, while other species decreased GC content despite all the species in these genera having GC-rich preferred codons (68% to 100% GC3 at preferred codons). 

## 4. Discussion

### 4.1. Variation in CUB in Diatoms

Our metanalysis showed striking variation of CUB in diatoms. Most diatom strains have GC-rich preferred codons, but few have AT-rich ones, and the switches between AT- and GC-rich preferred codons can occur within a single diatom genus (e.g., *Chaetoceros*). This lack of phylogenetic clustering of strains with similar sets of preferred codons suggests frequent shifts in preferred codon usage. Such CUB divergence was apparent in previous transgenic work in diatoms that required the transformed genes to be codon-adapted to the particular diatom species to ensure effective gene expression [49]. However, the extent and variation in CUB across diatoms has not been systematically described previously. The divergence in CUB within a genus, including switches between AT- and GC-rich preferred codons, was also reported in *Drosophila*, where *D. willistoni* has AT-rich preferred codons, while 11 other *Drosophila* species analysed have GC-rich preferred codons [13]. How and why such dramatic shifts in CUB occur remains unclear.

In the case of diatoms, the extent of variation in CUB is not surprising given the time scale of diatom evolution. Fossil evidence suggests that diatoms originated during the Jurassic period of the Mesozoic Era, about 150 to 200 million years ago [50]. Current species may thus be separated by many millions of years. For example, pennate and centric lineages have diverged 90 million years ago. Given a short generation time, species within a genus may be separated by billions of generations, providing enough time for such slow processes as the evolution of codon bias or significant shifts in GC content to take place.

The analysis of sequence divergence revealed that synonymous divergence within diatom genera can reach fairly high values, such as 59% to 77% between *Chaetoceros* species. So high synonymous divergence is comparable to that between mammalian orders (e.g., synonymous divergence between humans and mice is ~47% [51]). At least partly, this high divergence within diatom genera is due to lack of morphological characters that would allow taxonomists to split the diatom genera into finer scale taxonomic groupings. Short generation time must have also caused faster sequence divergence in diatoms compared to species with long generation time, such as metazoans. In the lab, many diatom species can reproduce at a rate of one generation per day, but in nature the generation time is likely to be longer. We can reasonably assume that wild diatom populations may go through at least several dozen generations per year. Following the equation *dS* = 2*tµ* [52], where *µ* is the mutation rate and *t* is the divergence time in generations, and assuming the mutation rate in diatoms is similar to that in *P. tricornum µ* ~ 4.77 × 10^−10^ [42], it is possible to estimate the divergence time between the strains. Given the *dS* between species within *Chaetoceros, Thalassiosira* and *Pseudo-nitzschia* genera is about 70%, the species in these genera may have diverged roughly 750 million generations ago. The average divergence in genus *Skeletonema* is lower, *dS* = ~15% (Figure 1), which corresponds to a divergence time of about 150 million generations. Assuming ~100 generations per year, we can estimate that the species we analysed in *Chaetoceros, Thalassiosira* and *Pseudo-nitzschia* genera diverged roughly 7.5 million years ago, while *Skeletonema* species diverged roughly 1.5 million years ago. However, according to fossil record, these genera appeared much earlier. First fossil evidence of *Chaetoceros* was found in the Kamchatka peninsula (Russia) and dated to 48–40 MY [53], with several high species diversification periods across the Eocene/Oligocene and Oligocene/Miocene boundaries [54]. According to the fossil database from Macquarie University (http://fossilworks.org), *Pseudo-nitzschia* appeared ~23 MY ago. The divergence between extant species is thus much more recent compared to the appearance time of the genera in the fossil record, which is likely due to species turnover and repeated bouts of species radiations and extinctions, as reported for other plankton including coccolithophores [55], diatoms [56], dinoflagellates [57] and planktic foraminifera [58].

### 4.2. What are the Causes of CUB Variation in Diatoms?

The differential evolution of CUB is thought to be driven by an interplay of neutral processes (i.e., mutational biases) and selection for optimal codons [1]. First, mutations are usually biased toward AT nucleotides, the well-known mutational bias (see Table 2 in [15]). Out of all the direct spontaneous mutation rate analyses for a range of species, only few are not biased toward AT and they belong to bacteria (see Table 2 in [15] and Table S12 in [14]). In diatoms, the only directly measured mutation rate is available for *P. tricornutum* [42]. In that study, the mutation rate of GC to AT nucleotides was ~2.21 higher than the mutation rate in the opposite direction. The extent of AT-mutation bias is very variable even between relatively closely related species (see Table 2 in [15] and Table S12 in [14]). In land plants for example, the GC to AT over AT to GC mutation rates can vary at least two-fold: ~3 in *Silene latifolia* [59] and ~6 in *Arabidospsis thaliana* [16]. Similarly, insects also show a wide variation in the extent of mutational AT-bias, ranging from ~4 in *D. melanogaster* [60] and *Chironomus riparius* [61], to ~11 in *Bombus terrestris* [62]. The most dramatic variation in AT-bias of mutations is observed in bacteria, where it can be absent (e.g., in *Burkholderia cenocepacia* and *Deinococcus radiodurans*) or really strong such as in the case of *Mesoplasma florum* with a GC to AT over AT to GC mutation rates ratio of ~16. Furthermore, the mutation spectrum and rate can vary depending on the environment [63]. Thus, plankton species may exhibit strong variation in mutation rate and spectrum [14,64,65,66,67], and extending the results of the only direct analysis of mutations in diatoms (in *P. tricornutum* [42]) to other species has to be done with caution. The mutation spectrum variation, both in bias strength and direction, may cause a shift in the mutation-drift balance and affect codon usage [68].

Selection acts on a mutation depending of its effect, if the change is toward or away from a preferred or an unpreferred codon. The strength of selection toward optimal codons can be quantified by *S*—the population-scaled selection coefficient, while the strength of CUB can be measured with ENC. *S*, and ENC of highly expressed genes are very variable between diatom species, with *S* ranging from −0.88 to 0.91, and ENC ranging from 44.28 to 57.27. The CUB and intensity of selection are thus very variable depending on diatom species. The diatom strain with lowest ENC (~44) is *Chaetoceros brevis* CCMP164 (MMETSP1435) isolated in the Antarctic Ocean. Strong codon bias of this species may reflect its large effective population size, rendering selection for CUB very effective. The genus *Chaetoceros* is indeed one of the most successful and widespread diatoms genera, followed by *Fragilariopsis*, *Thalassiosira*, and *Corethron* [23]. Interestingly, the species *Thalassiosira* FW (MMETSP1059), isolated in fresh water, shows one of the strongest CUB (ENC = 49) and highest selective coefficients (*S* = 0.67) for highly expressed genes. This indicates that freshwater diatom species can have effective populations comparable to those of marine species.

Our estimates of the selection coefficient based on the approach of Bulmer [2] and Sharp and colleagues [10] have to be taken with caution as this approach assumes that the species are at mutation-selection-drift equilibrium—an assumption that is clearly violated in most of the diatom species analysed (Table 2). Restricting the analyses to amino acids that did not change the preferred codon within a genus are likely more reliable. Unfortunately, the DNA polymorphism-based approaches for estimation of *S* that do not assume mutation-selection-drift equilibrium [69] cannot be used for the dataset in hand as larger samples of strains within the same species are required. Moreover, the two calculations of *S* in *P. tricornutum* RCC2967 revealed a twofold difference between the methods, suggesting that we may be underestimating *S* for other diatoms where the direct measurements of mutational pattern are unavailable.

The peculiar lifecycles of diatom species, with occasional sexual stage followed by many generations of asexual reproduction, may possibly be another factor affecting the extent of codon bias. Asexual reproduction in diatoms typically results in ever diminishing cell size because the shell of the daughter cell has to fit inside the parental shell [70,71]. Cell size is restored when diatoms go through the sexual stage of lifecycle, though some species, such as *P. tricornutum*, apparently can indefinitely reproduce asexually. Variation in frequency of sexual reproduction between the diatom lineages may affect mutational patterns, if they differ between meiosis and mitosis. E.g., if most mutations during meiosis are from GC to AT, while at mitosis mutations in the opposite direction are more frequent, then predominantly asexual lifecycle would lead to overall mutational pattern to be GC-biased, which would gradually increase GC content. Furthermore, predominantly asexual lifecycles may lead to widespread linkage disequilibrium across the genome, reducing efficacy of natural selection [72] and thus limiting the extent of codon bias. However, it is possible that even rare recombination during occasional sexual stage may be sufficient to break down linkage disequilibrium (LD) in very large populations of marine plankton species, as was reported for coccolithophore *E. huxleyi* [46]. Unfortunately, not enough is known about population genetics of marine plankton generally [73] and diatoms specifically, to judge whether variation in lifecycle is a significant factor in evolution of codon bias.

Unreliable species assignments in the dataset may be another source of errors or biases in the strength of codon bias analysis, though the overall estimates of codon bias (e.g., ENC and GC3 values) are strain-specific and robust to species mis-assignments. Given our estimates of sequence divergence between the strains, the species assignments of some of the strains in the MMETSP dataset appear incorrect and have to be taken with caution. In particular, this is the case for the two strains of *C. neogracile* that cannot possibly belong to the same species given 70% sequence divergence between these strains. On the other hand, the *Chaetoceros* strains that are labelled as different species (*C. debilis* MM31A-1 and *C. brevis* CCMP164) are likely conspecific. Another noteworthy point regarding species assignment is the identity of the model diatoms *T. pseudonana* CCMP1335 [26], collected in 1958 from Moriches Bay (Long Island, New York). Alverson et al. [44] reported previously that this strain is likely to have originated from a freshwater diatom species. Consistent with this, *T. pseudonana* CCMP1335 is indeed very closely related to the freshwater species *Thalassiosira* FW (lowest divergence in our dataset, *dS* ~0.01). Furthermore, consistent with the previous studies [44,45], we find that genus *Thalassiosira* is likely polyphyletic.

### 4.3. Concluding Remarks

The most surprising finding of our study is, perhaps, an overall very modest CUB in most of the diatom species, despite that their astronomical population sizes are expected to render weak selection for preferred codons very efficient. However, relatively modest intra-specific diversity in the diatoms (this study) and other plankton species [46,47] suggest that effective population size in marine plankton species is relatively small compared to the census size. Nevertheless, even this relatively small effective population size (~5–10 million) should be sufficiently large for selection for preferred codons to overpower drift. Thus, an alternative explanation—frequent changes of the preferred codons, may be a more likely explanation for weak CUB in most diatom species. Indeed, we documented multiple shifts in the preferred codon usage, including such major shifts as the change from GC-rich to AT-rich preferred codons in some *Chaetoceros* species. Such shifts make the set of optimal codons a moving target that is never reachable by the slow process of CUB evolution.

## Figures and Tables

**Table 1:** Strength of selection only calculated with codons that did not show any change in preferred codon usage within a clade.

*Pseudo-nitschia*	*S*
MMETSP0327_*P.delicatissima*_B596	0.3224 ^***^
MMETSP0329_*P.arenysensis*_B593	0.5128 ^***^
MMETSP0853_*P.fraudulenta*_WWA7	0.1069 ^***^
MMETSP1060_*P.pungens*_cingulata	0.1282 ^***^
MMETSP1061_*P.pungens*_pungens	0.2265 ^***^
MMETSP1423_*P.heimii*_UNC1101	0.0491 ^***^
MMETSP1432_*P.delicatissima*_UNC1205	0.3986 ^***^
***Minutocellus***	
MMETSP1070_*M.polymorphus*_NH13	0.4625 ^***^
MMETSP1322_*M.polymorphus*_RCC2270	0.5208 ^***^
MMETSP1434_*M.polymorphus*_CCMP3303	−0.1424 ^ns^
***Leptocylindrus***	
MMETSP0321_*D.danicus*_B650	0.4990 ^***^
MMETSP0322_*D.danicus*_apora_B651	0.5163 ^***^
MMETSP1362_*D.danicus*_CCMP1856	0.7312 ^***^
***Thalassiosira***	
MMETSP0404_*T.rotula*_CCMP3096	0.5433 ^***^
MMETSP0494_*T.gravida*_GMp14c1	0.2919 ^***^
MMETSP0740_*T.minuscula*_CCMP1093	0.2284 ^***^
MMETSP0881_*T.weissflogii*_CCMP1336	0.7675 ^***^
MMETSP0905_*T.antarctica*_CCMP982	0.7422 ^***^
MMETSP0913_*T.rotula*_GSO102	0.6406 ^***^
MMETSP0973_*T.oceanica*_CCMP1005	0.4032 ^***^
MMETSP1059_*T*._FW	0.5631 ^***^
MMETSP1067_*T.punctigera*_Tpunct2005C2	0.5752 ^***^
MMETSP1071_*T*._NH16	0.6420 ^***^
MMETSP1422_*T.weissflogii*_CCMP1010	0.6491 ^***^
***Skeletonema***	
MMETSP0013_*S.costatum*_RCC1716	0.4699 ^***^
MMETSP0319_*S.marinoi*_SM1012Hels	0.5485 ^***^
MMETSP0320_*S.marinoi*_SM1012Den	0.5096 ^***^
MMETSP0563_*S.dohrnii*_SkelB	0.4569 ^***^
MMETSP0578_*S.grethae*_CCMP1804	0.3390 ^***^
MMETSP0593_*S.japonicum*_CCMP2506	0.3625 ^***^
MMETSP0604_*S.menzellii*_CCMP793	0.4057 ^***^
MMETSP0920_*S.marinoi*_SkelA	0.4275 ^***^
MMETSP1039_*S.marinoi*_FE7	0.3182 ^***^
MMETSP1040_*S.marinoi*_FE60	0.2763 ^***^
MMETSP1428_*S.marinoi*_UNC1201	0.5165 ^***^

*** means significantly high *S* compared to the rest of the genome according to Sharp and colleagues [10] method and *ns* means not significant.

**Table 2:** Evolution of GC content at 3rd position of synonymous codons in the 4 diatom genera with >7 strains analysed.

Genus	Species	GC3 of Preferred Codons	To Preferred	To Unpreferred	Total SUBSTITUTIONS	GC Ancestral	GC Current	2 × 2 *χ*^2^ Test
*Chaetoceros* (53 genes; total length 77,214 nucleotides)	MMETSP0092_C.affinis_CCMP159	0.80	1524	2348	3872	36.39	52.22	0.0001
	MMETSP0150_C.debilis_MM31A	0.64	1906	745	2651	26.10	68.73	0.0001
	MMETSP0200_C.GSL56	0.65	4609	2745	7354	27.89	55.22	0.0001
	MMETSP0754_C.neogracile_CCMP1317	0.15	2713	2257	4970	50.99	33.02	0.0001
	MMETSP1336_C.neogracile_RCC1993	0.61	4727	2302	7029	24.58	57.01	0.0001
	MMETSP1429_C.UNC1202	0.68	4671	1957	6628	21.23	70.70	0.0001
	MMETSP1435_C.brevis_CCMP164	0.70	1099	531	1630	22.09	75.28	0.0001
	MMETSP1447_C.dichaeta_CCMP1751	0.10	3795	3405	7200	46.01	48.17	0.0001
*Pseudo-nitzschia* (1094 genes; total length 2,006,448 nucleotides)	MMETSP0142_P.australis_10249	1.00	58,578	74,556	133,134	59.37	47.40	0.0001
	MMETSP0327_P.delicatissima_B596	0.85	34,900	25,100	60,000	45.42	60.73	0.0001
	MMETSP0329_P.arenysensis_B593	0.80	46,274	50,697	96,971	55.36	48.87	0.0001
	MMETSP0853_P.fraudulenta_WWA7	1.00	46,621	29,852	76,473	44.89	62.85	0.0001
	MMETSP1060_P.pungens_cingulata	1.00	58,723	16,794	75,517	25.02	83.10	0.0001
	MMETSP1061_P.pungens_pungens	1.00	58,414	16,385	74,799	24.72	83.46	0.0001
	MMETSP1423_P.heimii_UNC1101	1.00	76,249	75,362	151,611	50.88	52.37	0.0013
	MMETSP1432_P.delicatissima_UNC1205	0.87	23,380	27,853	51,233	46.63	60.20	0.0001
*Skeletonema* (125 genes; total length 140,730 nucleotides)	MMETSP0563_S.dohrnii_SkelB	0.64	493	463	956	44.56	49.27	0.0185
	MMETSP0578_S.grethae_CCMP1804	0.67	1704	861	2565	26.82	65.50	0.0001
	MMETSP0593_S.japonicum_CCMP2506	0.64	1466	688	2154	24.56	72.93	0.0001
	MMETSP0604_S.menzellii_CCMP793	0.64	2935	2354	5289	41.96	51.35	0.0001
	MMETSP1040_S.marinoi_FE60	0.64	1220	907	2127	38.81	61.64	0.0001
	MMETSP1428_S.marinoi_UNC1201	0.64	2122	1728	3850	40.44	54.10	0.0001
*Thalassiosira* (204 genes; total length 398,550 nucleotides)	MMETSP0404_T.rotula_CCMP3096	1.00	4506	2778	7284	58.83	61.86	0.0001
	MMETSP0494_T.gravida_GMp14c1	1.00	3225	3036	6261	64.29	54.72	0.0008
	MMETSP0740_T.minuscula_CCMP1093	0.96	6697	7244	13,941	49.11	55.70	0.0001
	MMETSP0905_T.antarctica_CCMP982	0.97	5058	8047	13,105	70.67	38.99	0.0001
	MMETSP0973_T.oceanica_CCMP1005	1.00	17,637	11,133	28,770	50.84	63.00	0.0001
	MMETSP1067_T.punctigera_Tpunct2005C2	1.00	13,348	5128	18476	39.15	73.28	0.0001
	MMETSP1071_T.NH16	1.00	10,106	7236	17,342	52.98	59.81	0.0001
	MMETSP0881_T.weissflogii_CCMP1336	1.00	10,543	10,658	21,201	55.92	51.45	0.2682
	MMETSP1059_T. FW	0.68	11,369	10,174	21,543	65.27	38.67	0.0001

The numbers of substitutions are counted for the external branches of the phylogenies shown in Figure 3. To avoid having too few data because of short species branches (that counted only a few hundred substitutions), we excluded some strains to have only one individual per species, particularly in *Skeletonema* and *Thalaisiosira*. *Total substitutions* is the total synonymous substitutions in the species branch; *GC ancestral* is the ancestral GC content of the third position before the substitution from the branch leading to the species; *GC current* is the current GC content of the third position of *Total substitutions* sites; 2 × 2 *χ*^2^ tests significance of difference in numbers of *To preferred* and to *To unpreferred* according to the Table S2.

## Supplementary Materials

The following are available online at https://www.mdpi.com/2073-4425/10/11/894/s1, Table S1: Origin and taxonomy and the 83 diatom strains; Table S2: GC3, ENC and FOP indexes calculated by codonW for each strain analysed; Table S3: Optimal codons noted with * of each strains; Table S4: Selective coefficient values and GC3 of preferred codons; Table S5: Synonymous divergence within *Pseudo-nitzschia*, *Skeletonema*, *Thalassiosira* and *Chaetoceros* genus; Figure S1: Distribution of the different codon analysis indexes in each species; Figure S2: Phylogenetic tree of all strains built by STAG; and Figure S3: DensiTree including all species (except MMETSP0919 because of lot of missing data) from PRANK analysis.
