# Supplementary material for: Evolution of Codon Usage Bias in Diatoms
Source: Genes (Basel). 2019 Nov 6;10(11):894. doi: 10.3390/genes10110894 (PMC6896221; doi:10.3390/genes10110894)
Supplement: Supplementary file 1 [file genes-10-00894-s001.zip › Figure_S3.pdf]

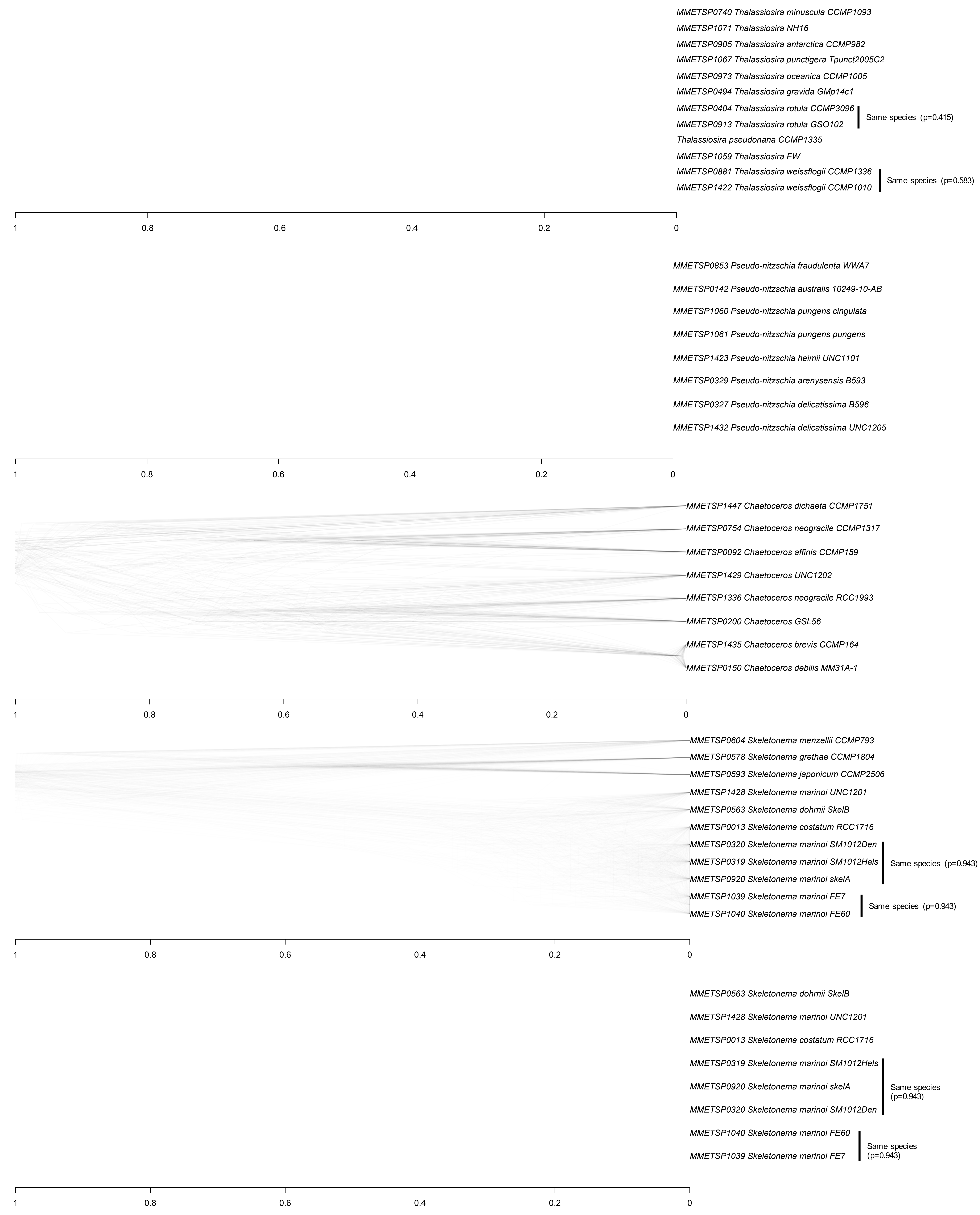
MMETSP0740 Thalassiosira minuscula CCMP1093
MMETSP1071 Thalassiosira NH16
MMETSP0905 Thalassiosira antarctica CCMP982
MMETSP1067 Thalassiosira punctigera Tpunct2005C2
MMETSP0973 Thalassiosira oceanica CCMP1005
MMETSP0494 Thalassiosira gravida GMp14c1
MMETSP0404 Thalassiosira rotula CCMP3096
MMETSP0913 Thalassiosira rotula GSO102
Same species (p=0.415)
Thalassiosira pseudonana CCMP1335
MMETSP1059 Thalassiosira FW
MMETSP0881 Thalassiosira weissflogii CCMP1336
MMETSP1422 Thalassiosira weissflogii CCMP1010
Same species (p=0.583)
1
0.8
0.6
0.4
0.2
0
MMETSP0853 Pseudo-nitzschia fraudulenta WWA7
MMETSP0142 Pseudo-nitzschia australis 10249-10-AB
MMETSP1060 Pseudo-nitzschia pungens cingulata
MMETSP1061 Pseudo-nitzschia pungens pungens
MMETSP1423 Pseudo-nitzschia heimii UNC1101
MMETSP0329 Pseudo-nitzschia arenysensis B593
MMETSP0327 Pseudo-nitzschia delicatissima B596
MMETSP1432 Pseudo-nitzschia delicatissima UNC1205
1
0.8
0.6
0.4
0.2
0
MMETSP1447 Chaetoceros dichaeta CCMP1751
MMETSP0754 Chaetoceros neogracile CCMP1317
MMETSP0092 Chaetoceros affinis CCMP159
MMETSP1429 Chaetoceros UNC1202
MMETSP1336 Chaetoceros neogracile RCC1993
MMETSP0200 Chaetoceros GSL56
MMETSP1435 Chaetoceros brevis CCMP164
MMETSP0150 Chaetoceros debilis MM31A-1
1
0.8
0.6
0.4
0.2
0
MMETSP0604 Skeletonema menzellii CCMP793
MMETSP0578 Skeletonema grethae CCMP1804
MMETSP0593 Skeletonema japonicum CCMP2506
MMETSP1428 Skeletonema marinoi UNC1201
MMETSP0563 Skeletonema dohrnii SkelB
MMETSP0013 Skeletonema costatum RCC1716
MMETSP0320 Skeletonema marinoi SM1012Den
MMETSP0319 Skeletonema marinoi SM1012Hels
MMETSP0920 Skeletonema marinoi skelA
Same species (p=0.943)
MMETSP1039 Skeletonema marinoi FE7
MMETSP1040 Skeletonema marinoi FE60
Same species (p=0.943)
1
0.8
0.6
0.4
0.2
0
MMETSP0563 Skeletonema dohrnii SkelB
MMETSP1428 Skeletonema marinoi UNC1201
MMETSP0013 Skeletonema costatum RCC1716
MMETSP0319 Skeletonema marinoi SM1012Hels
MMETSP0920 Skeletonema marinoi skelA
MMETSP0320 Skeletonema marinoi SM1012Den
Same species (p=0.943)
MMETSP1040 Skeletonema marinoi FE60
MMETSP1039 Skeletonema marinoi FE7
Same species (p=0.943)
1
0.8
0.6
0.4
0.2
0
