# Supplementary figures and images for: Evolution of Codon Usage Bias in Diatoms
Source: Genes (Basel). 2019 Nov 6;10(11):894. doi: 10.3390/genes10110894 (PMC6896221; doi:10.3390/genes10110894)

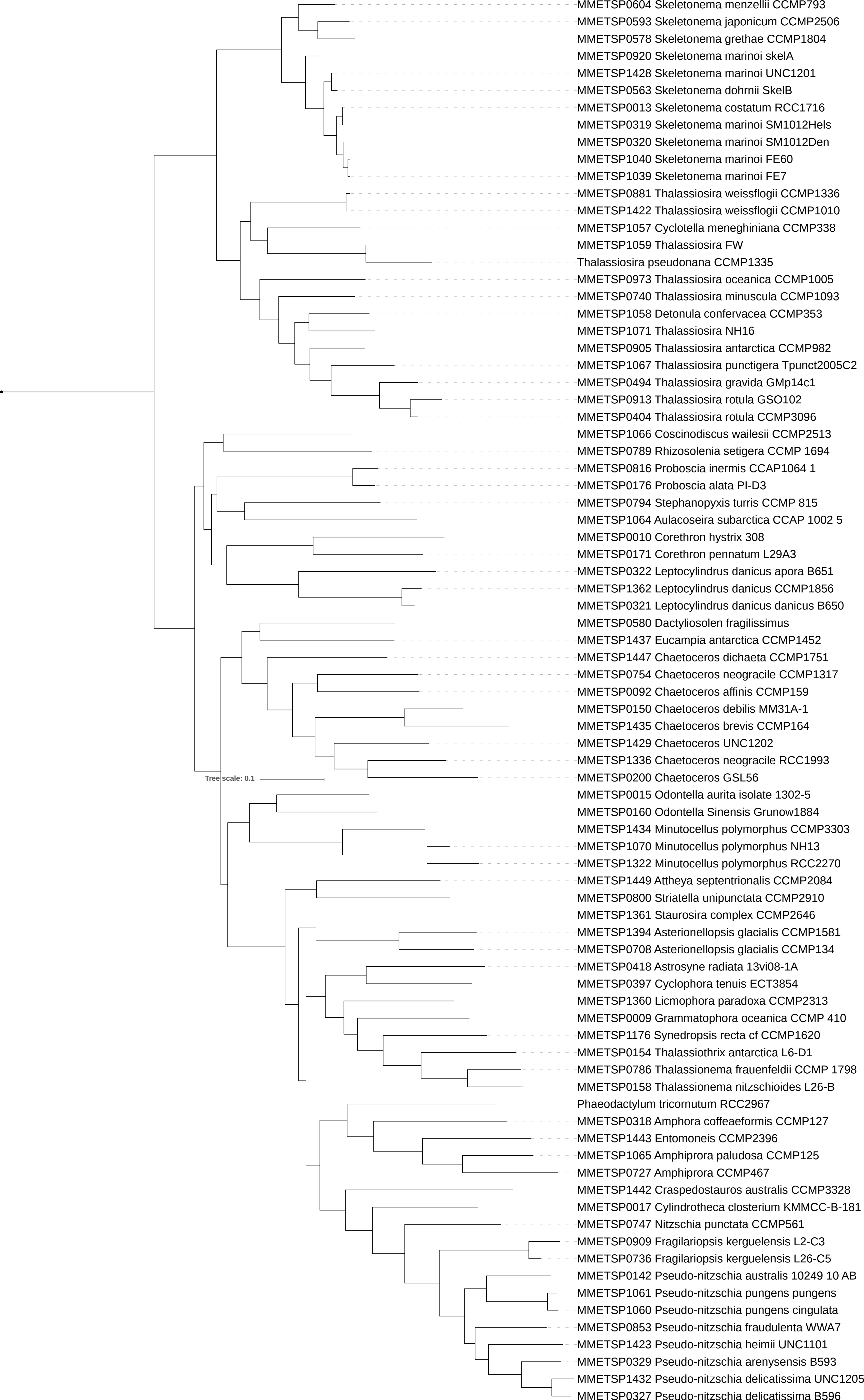

Supplement: Supplementary file 1 [file genes-10-00894-s001.zip › Figure_S2.pdf]

GC at GC3s sites

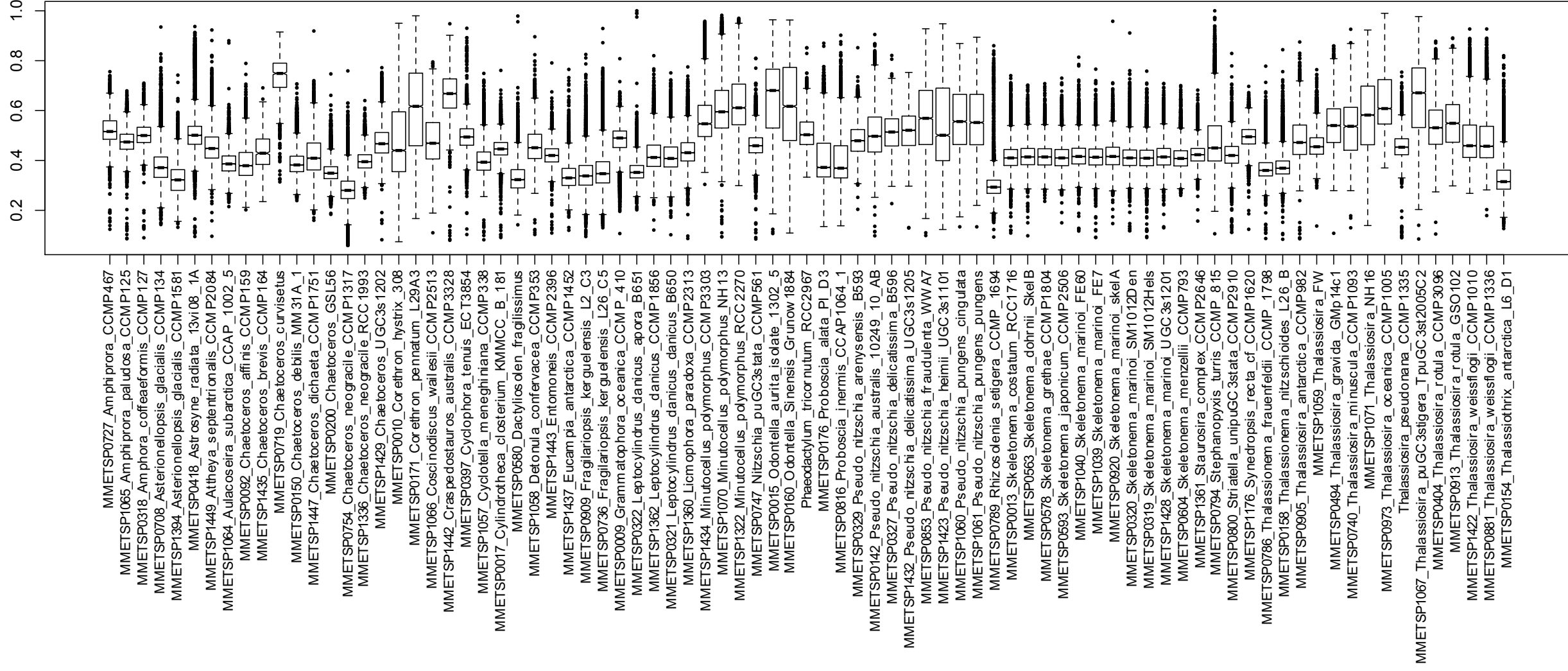

Frequency of optimal codons

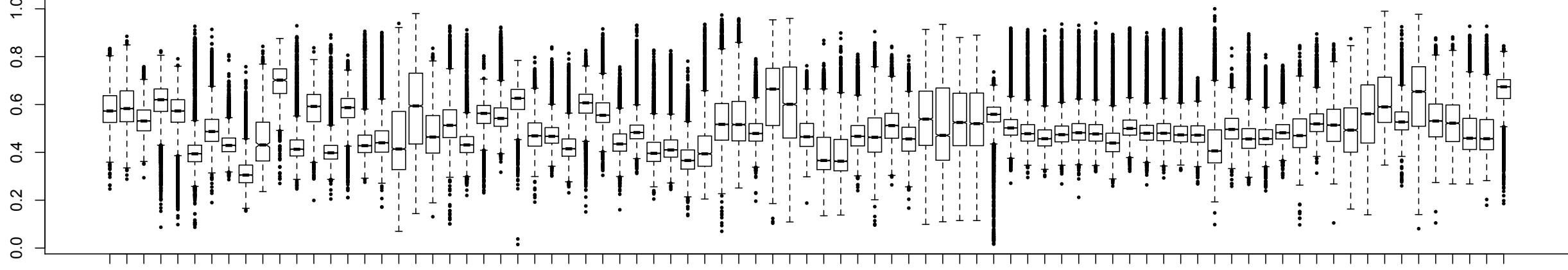

Effective number of codons

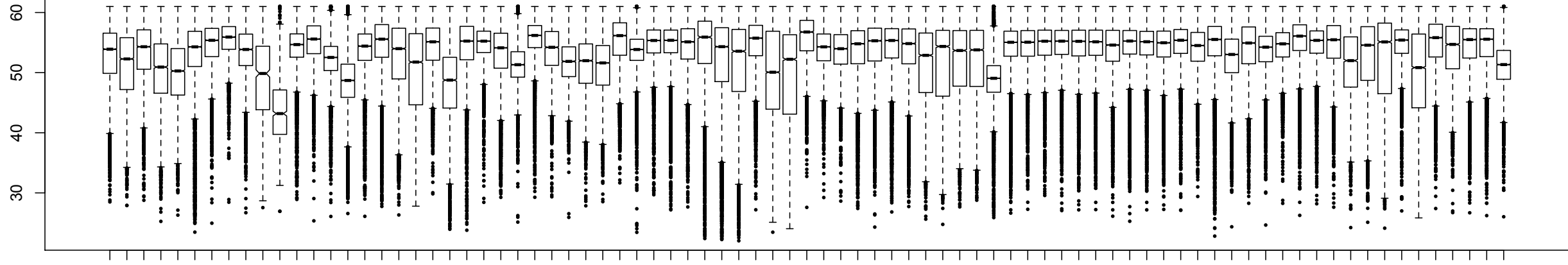

Supplement: Supplementary file 1 [file genes-10-00894-s001.zip › Figure_S1.pdf]
